# Long-Term Effects of Xerophytic Shrub *Haloxylon ammodendron* Plantations on Soil Properties and Vegetation Dynamics in Northwest China

**DOI:** 10.1371/journal.pone.0168000

**Published:** 2016-12-16

**Authors:** Baoli Fan, Aiping Zhang, Yi Yang, Quanlin Ma, Xuemin Li, Changming Zhao

**Affiliations:** 1 State Key Laboratory of Grassland Agro-Ecosystems, School of Life Sciences, Lanzhou University, Lanzhou, Gansu, P. R. China; 2 State Key Laboratory of Desertification and Aeolian Sand Disaster Combating, Gansu Desert Control Research Institute, Lanzhou, Gansu, P. R. China; University of Vigo, SPAIN

## Abstract

The xerophytic desert shrub *Haloxylon ammodendron* (C. A. Mey.) Bunge. is distributed naturally in Asian and African deserts, and is widely used for vegetation restoration in the desert regions of Northern China. However, there are limited long-term chrono-sequence studies on the impact of changed soil properties and vegetation dynamics following establishment of this shrub on mobile sand dunes. In Minqin County, Gansu Province, we investigated soil properties and herbaceous vegetation development of 10, 20, 30, 40, 50-year-old *H*. *ammodendron* plantations on mobile sand dunes. Soil sampling at two depths (0–5 and 5–20 cm) under the shrubs determined SOC, nutrition and soil physical characteristics. The results showed that: establishment of *H*. *ammodendron* had improved soil physio-chemical properties, increased thickness of soil crusts and coverage of biological soil crusts (BSCs), and promoted development of topsoil over an extended period of 5 decades. Soil texture and soil nutrition improved along the chrono-sequence according to three distinct phases: i) an initial fast development from 0 to 10 years, ii) a stabilizing phase from 10 to 30 years followed by iii) a relatively marked restoration development in 40 and 50-year-old plantations. Meanwhile, herbaceous community coverage also markedly increased in 30-year-old plantations. However, both soil and vegetation restoration were very slow due to low annual precipitation in Minqin county compared to other Northern China sand afforestation sites. Canonical Correspondence Analysis results demonstrated that herbaceous plant development was closely associated with changes in soil texture (increased clay and silt percentage) and availability of soil nutrients. Thus our results indicated that selection of the long-lived shrub *H*. *ammodendron* is an essential and effective tool in arid desert re-vegetation.

## Introduction

Desertification is considered one of the most critical ecological and environmental issues worldwide and has gained increasing attention [1] especially in China. In order to combat desertification, the Chinese government has been carrying out large-scale afforestation, following many studies that demonstrated that it is an effective and widely used management option to curb severe sand erosion [2]. However, the impact of plantations on soil characteristics and vegetation dynamics in these arid systems are poorly understood, relative to their importance for wind breaks and sand-fixation, especially when previous studies found that desert steppe ecosystem recovery is slower than the recovery reported in desert ecosystems elsewhere [3,4]. Moreover, short-term afforestation may show initial success in revegetation processes in arid regions, but its impact on biodiversity and its ability to achieve full ecological restoration will depend on the type of reforestation carried out and its suitability to the local environment [5–7]. For example, in parts of the MuUs Desert of Inner Mongolia Autonomous Region, a number of researchers reported successful afforestation in short-term studies, however tree mortality reached 70% after 20 years, consequently with renewed desertification, it erased the earlier reported gains. According to Cao [6] (2008) and Wu *et al*. [7] this loss of vegetation cover to lower levels prior to afforestation, exacerbated soil moisture loss, thus perpetuating the desertification. Unfortunately, most studies that test the adaptability of vegetation and its restoration impact at a given site are typically monitored for only short periods. For example, Wortley *et al*. [8] emphasized that the age groups of empirical assessments of restoration outcomes over 35 years old were just 5%. Consequently, the lack of long-term field research largely limits our understanding of ecological restoration effects across varied ecological regions. This affirms the necessity to conduct long-term field investigations on ecological restoration of desert steppe ecosystems.

Nevertheless, some previous short-term studies in northern China have estimated the influence of vegetation restoration age on soil physio-chemical properties in the semiarid Horqin sand lands (1–3 decades) [9–14] and revegetation with several shrub species on sand dunes in the transitional Shapotou area of Ningxia Province (< 34 years) [15–16]. There are only a few existing long-term studies that extend to 40 or 50 years, such as those by Li *et al*. [3,17–19] and Wang *et al*. [20]. The latter study, based in the Tengger Desert, stated that from the original 18 shrub species only 3 survived after 17 years, but importantly the aerial deposition of fine dust particles over 40 years facilitated by the shrubs, aided the restoration. According to Wang et al. [20] this resulted in a strengthening of bio-geochemical cycling processes and an accumulation of fine particles from the aeolian dust, which contributed to micro-biotic soil crust formation, leading to surface soil enrichment and the stabilization of sand dunes.

These studies point to the concerns in arid and semiarid regions that appropriate species selection is a key factor for longevity of environmental restoration [21, 22], but still little is known about the rehabilitation impact of establishing a single long-lived shrub species on shifting-sand-fixation in arid sandy landscapes. Especially about its effects on soil and vegetation composition, such as in Minqin County, Gansu Province [2], where the effects of soil and vegetation restoration rate may be different, compared to a relatively more humid semiarid desert area. Growth and survival of vegetation in China's arid and semiarid regions are strongly linked to available water [23], so it is vital to choose xerophytic desert shrubs with high tolerance to water stress and survival so that the primary focus is on sand-fixation through consistent and long-term aeolian dust capture and deposition and thus soil amelioration and ecological restoration. The xerophytic desert shrub *Haloxylon ammodendron* is distributing naturally in Asian and African deserts [24, 25], and is widely accepted for use in vegetation restoration in the desert regions of northern China because of its previous success in stabilizing sand dunes. For example, an ongoing restoration project that is expanded every year in Minqin County, has a dominant plant community of *H*. *ammodendron*, which covers about 0.035 million hectares (51.5%) of the restoration area [26]. Due to this widespread use of *H*. *ammodendron*, it is critical to understand the restoration processes and the long-term ecological effects on soil and vegetation. Here, plantations of *H*. *ammodendron* range from 10 to 50 years old giving a unique opportunity to study the effect of shrub afforestation on soil properties and vegetation communities over a long period in the Minqin Desert region. The objectives of our study were: (1) to assess the impacts of long-term establishment of *H*.*ammodendron* in terms of soil physio-chemical properties and vegetation dynamics; (2) to determine whether the restoration of soil and vegetation in the Minqin desert is different to published data from other desert regions in north China; and (3) to provide practical suggestions for shrub plantation management and ecological restoration.

## Materials and Methods

### Ethics statement

No specific permits were required for the described field studies, because *H*. *ammodendron* is not an endangered or protected species. Collecting soils and plant samplings from each *H*. *ammodendron* plantations did not involve any National Nature Reserve in China. Additionally, this study was conducted at Minqin Desert Control Station, an experimental base belonging to the Lanzhou University and the Gansu Desert Control Research Institute.

### Site description

The study area lies in Minqin County, on the lower reaches of Shiyang River at the eastern end of the Hexi Corridor, Gansu Province, northwest China (101°59′E-104°12′E, 38°08′N-39°26′N) [27]. This area is subjected to an arid desert climate with an average annual temperature of 7.8°C. The average annual precipitation is 116.5 mm, with average annual potential evaporation of 2383.7 mm [28]. The mean wind speed is 2.4 m.s^-1^ and the average number of days with gales (wind velocity ≥ 17 m.s^-1^) is 27.4 days per year. Sand and gravel deserts, salt marshes and low mountains account for 94.2% of the region, with irrigated oasis areas making up the remainder of the region. Minqin County is surrounded by the Badain Jaran Desert in the northwest and the Tengger Desert in the east and the detailed map of the study area could be seen in Dong et al. [29]. The zonal soil type is part aeolian, part irrigated desert soils and grey brown desert soil [30] (OSU SCAS, 2004). During the process of sand dune stabilization, these desert soils can form a physical crust consisting of increased clay and silt fractions, largely from capture and deposition of aeolian particles by shrubs. In turn a biological soil crust (BSC) can form from the deposition of attached nutrients and algal growth, and the role of physical crust is different from BSCs. Most trees and shrubs in this area are from plantation activities consisting of native xerophytic shrubs, small shrubs and herbaceous plants. The vegetation ground cover is generally less than 15% and this percentage cover continues to degrade. Precipitation is usually the only source of water for desert plant growth. The fertility in all soil types in this area is very low due to the arid climate and sparse desert vegetation.

*H*. *ammodendron* is a chenopodiaceae shrub, which is widely distributed with deep-root system and native to Central Asian with a longevity and that could exceed one hundred years. In the Minqin area, natural shrub vegetation still exists, but plantations are generally used in vegetation reestablishment programs to stabilize shifting sand dunes. Consequently *H*. *ammodendron* as 2-year-old seedlings, at a density of 16.5 individuals per 100 m^2^, were planted in mobile sand dunes each decade since the 1960s. It was here that clearly distinguishable age trends were identified in the decade-old plantations. Bound straw in a checkerboard layout (1 m^2^) by soil sealing was used to protect seedlings from strong wind blown sand and to assist in dune stabilization. Over the years, the straw was not repaired, but allowed to decay or be blown away.

### Vegetation survey

During early September, 2013, a total of eighteen 20×20 m2 plots were selected at random on the windward sides of dunes with similar slopes. Plots were equally distributed in each of the five decadal groups of *H*. *ammodendron* established in the 1960’s, 1970’s, 1980’s, 1990’s and 2003, and in a nearby mobile sand dune, served as the control site (CK). Shrub height, basal diameter and ground coverage of *H*. *ammodendron* were recorded for each plot. The *H*. *ammodendron* leaf is branchlet with reduced leaves therefore the length and diameter of the branchlets were measured with a micrometer. Thus the leaf area (LA) was calculated as: *LA* = *L*_*n*_ × *D* × *π*, where; *L*_n_ is the leaf length, and *D* is the leaf diameter. Five 1 x 1 m^2^ quadrats were randomly selected within each plot, where herbaceous species composition, number, height and coverage of plants in each quadrat were recorded. In the text the shrubs *Calligonum mongolicum* and *Reaumuria songarica* were naturally occurring however these were sparsely scattered across the stabilized sand dune and thus only the most dominant or changed herbaceous species were included in this study. Native species diversity in plant communities was determined by two diversity indices: the Shannon–Wiener index and the Simpson index.

1. Simpson index (*D*): $D=1-\sum_{i=1}^{s}N_{i}^{2}$
2. Shannon–Wiener index (*H*): $H=-\sum_{i=1}^{s}N_{i}\text{ ln }N_{i}$

Where; *s* is the species number in the community, and *Ni* is the relative importance value of species *i* in each plot, which was calculated from the mean of relative cover, height and relative abundance [31].

### Soil sampling and measurements

Soil crust thickness and its percentage area within each quadrat were determined immediately after herbaceous cover was measured at the same location. The biological soil crusts (BSCs), which were distinguishable by visible differences in the topsoil colour and soil crust thickness within each quadrat, were quantified with 0-200mm electronic Vernier calipers, whilst BSCs percentage area was determined using the method described by Li *et al*. [19]. Within each *H*. *ammodendron* plot and the control, five sampling ditches were dug, from where soil samples were collected manually from soil depths of 0–5 cm and 5–20 cm. Stainless steel cylinders (100 cm^3^ in volume) were used to sample soil for bulk density and water content determination. The soil water content at 0–5 and 5–20 cm depths was determined gravimetrically on an oven dry mass basis after drying samples at 105°C for 24 h. Additional soil samples for particle size distribution (PSD) determination were air dried in the laboratory, after which gravel and roots in the samples were carefully removed. PSD was determined by laser diffraction equipment (Mastersizer 2000, UK) and divided into four particle sizes: clay content (<0.002 mm), silt content (0.002~0.02 mm), fine sand (0.02~0.2 um) and coarse sand (0.2~2 mm).

Further air-dried soil samples were ground in an agate mortar and passed through a 1 mm sieve to determine pH, EC, total P and available P, and 0.25mm sieve to determine Soil Organic Carbon (SOC) and total N. Soil pH was determined from 1:1 soil water solutions and electrical conductivity (EC) determined using 1:5 soil-water aqueous extracts. Total SOC was determined by the Walkley-Black method and a factor of 1.3 was used to determine organic C recovery [32]. Total N was determined using semi-micro Kjeldahl digestion followed by distillation and titration using a Kjeltec auto distillation–titration unit [33]. Soil samples for total phosphorus (Total P) was digested by HClO_4_-H_2_SO_4_ [34] and determined by molybdenum-blue colorimetry. Available phosphorus (Available P) was determined by the Bray method [35].

### Statistical analysis

Differences in development of *H*. *ammodendron* (height, basal diameter, coverage and leaf area), herbaceous species diversity indices and coverage among plantation ages, measured soil parameters among plantation ages and soil layers were evaluated using analysis of variance (ANOVA) followed by multiple range test (Tukey test). Significant difference between groups were identified taking *p*≤0.05 as significant. Data were log transformed when necessary to meet assumptions of ANOVA for normality and homogeneity of variance. Statistical analysis of the above data was achieved with SPSS 16.0 software. Canonical Correspondence Analysis (CCA)from the CANCOR package in R (R Foundation for Statistical Computing ver 3.2.4) was used to analyse the relationships amongst soil properties (clay content, silt content, fine sand, coarse sand, soil water, bulk density, pH, EC, SOC, Total N and SP), native herbaceous distribution and vegetation composition (the relative abundance) relative to the soil variables and *H*. *ammodendron* plantation age. Total P were not used in CCA due to almost no significant changes among the different groups.

## Results

### Development of *H*. *ammodendron* plantations

**Fig 1:**
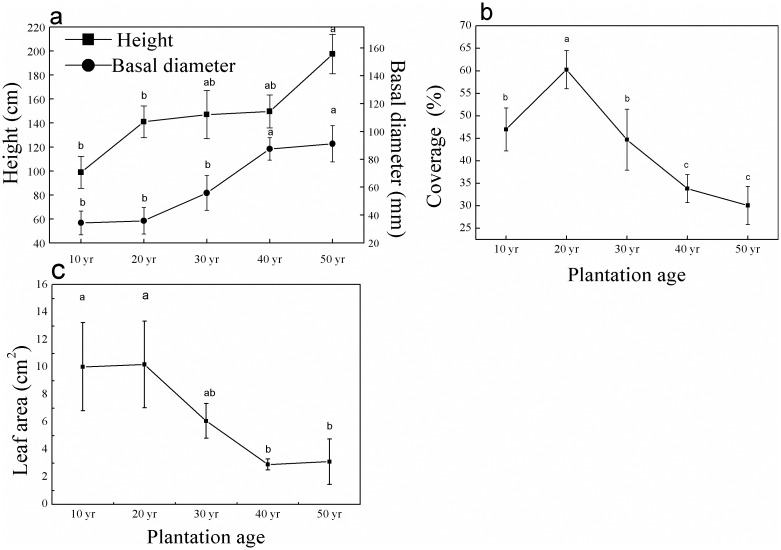
Changes in a) height and basal diameter, b) cover and c) leaf area (mean±SE) of *H*. *ammodendron* according to plantation age (Different letters above the error bars designate significant differences at *p*<0.05 between plantation ages).

Generally, mean height and basal diameter of the individual shrubs increased with plantation age (Fig 1a). Shrub height developed quickly during the first 20 years, however growth rate tended to stabilize during the 20–40 year period, but growth was not significantly different between ages. However, shrub height was significantly higher at 50 years (197.5 cm) than that of 10 (98.8 cm) and 20-year (141 cm) plots. Conversely, basal diameter developed quickly during the 20–40 year period, with an average growth rate of 50% per decade, but no significant change was detected between 10 and 20 years plots (Fig 1a). In the initial decades, *H*. *ammodendron* coverage peaked at 60.2% (Fig 1b), then continually decreased in each following decade to 30.1% in the 50-year-old plots. The leaf area developed quickly during the first 20 years and peaked at 10.0 cm^2^ in 20-year plots, then continually decreased each decade to 3.1 cm^2^ in the 50-year-old plots (Fig 1c).

### Changes in soil physical properties of *H*. *ammodendron* plantations

**Fig 2:**
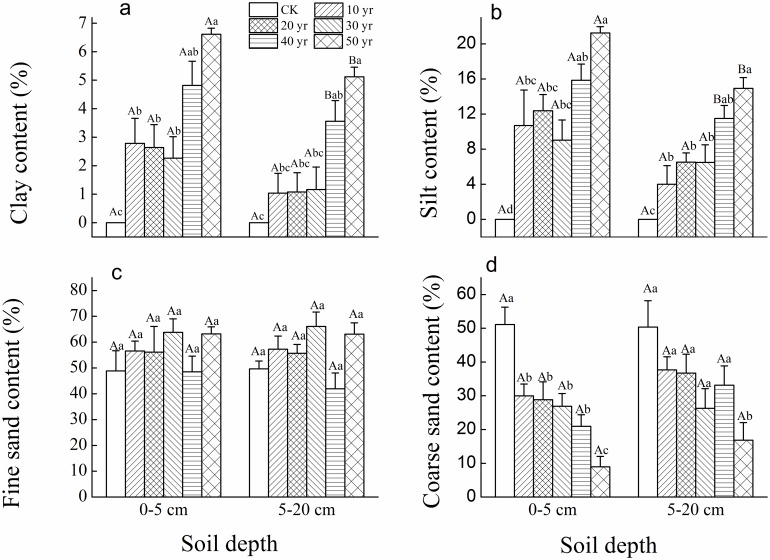
Changes in soil particle size distribution (%, mean±SE) at 0–5 cm and 5–20 cm soil depths in plantations of different ages and mobile sand dune (CK). (Different uppercase letters indicate significant differences in soil properties between two soil depths in the same plantation age at *p*<0.05. Different lowercase letters indicate significant differences in soil properties among different plantation ages with the same soil depth at *p*<0.05).

According to the PSD data, mobile sand dunes had the least clay and silt contents nearing 0%, but had the largest coarse sand content (50%) in comparison to all 5 decadal plantations (Fig 2). In the topsoil (0-5cm) and sub-topsoil (5-20cm), the differences in PSD (except for fine sand content) between mobile sand dune and 10-year-old plantations were significant, while the increase in clay and silt content among 10, 20, and 30-year-old plantations was not significant (Fig 2a and 2b). Whereas there was a marked increase in clay and silt content at both depths but differences were only significant between 50-year-old and 10 to 30-year-old plantations. Meanwhile, the clay and silt content was significantly less at the lower depths in the two oldest plantations, but nevertheless, larger than earlier decades and significantly in 50-year-old sites (Fig 2a and 2b). Additionally there was no significant variation in fine sand content among the groups (Fig 2c). Conversely coarse sand significantly decreased at both depths in all decades compared to CK, especially in the 50-year-old sites (Fig 2d).

**Fig 3:**
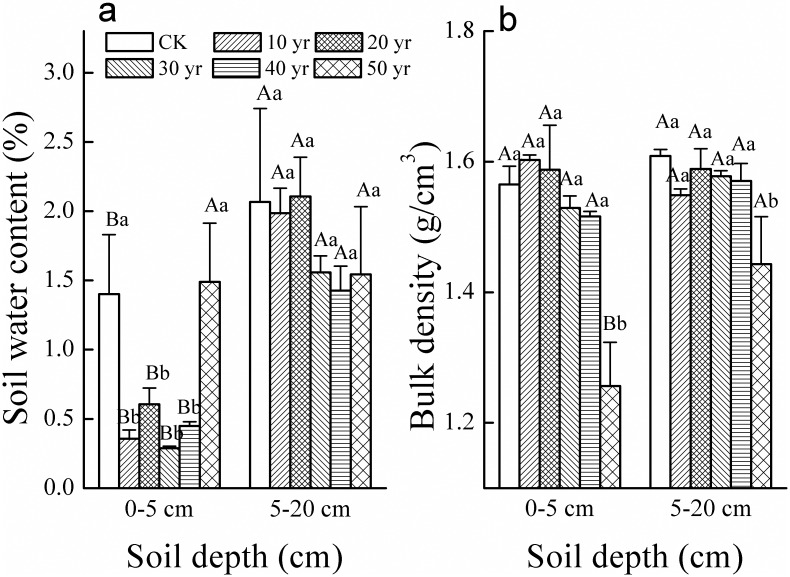
Soil water content (a) and bulk density (b) (mean±SE) in the 0–5 cm and 5–20 cm soil layers in plantations established each decade for 50 years (CK-mobile sand dune). (Different uppercase letters indicate significant differences in soil properties between two soil depths in the same plantation age at *p*<0.05. Different lowercase letters indicate significant differences in soil properties among different plantation ages with the same soil depth at *p*<0.05).

The water content for all sites was significantly lower in the topsoil layer by 1–1.5% compared to the subsurface soil (5-20cm) except at the 50-year-old site (Fig 3a). Meanwhile, topsoil (0-5cm) water content in the four youngest plantations was significantly less than CK and the 50-year-old site. Subsurface soil water content was not significantly different among sites (Fig 3a). Bulk density (at 1.55 g.cm^-3^) did not show significant variations among sites in the first four decades but was significantly reduced to less than 1.3 g.cm^-3^ in the 50-year-old sites in topsoil and to ~1.45 g.cm^-3^ in subsurface soil (Fig 3b).

### Changes in soil chemical properties of *H*. *ammodendron* plantations

**Fig 4:**
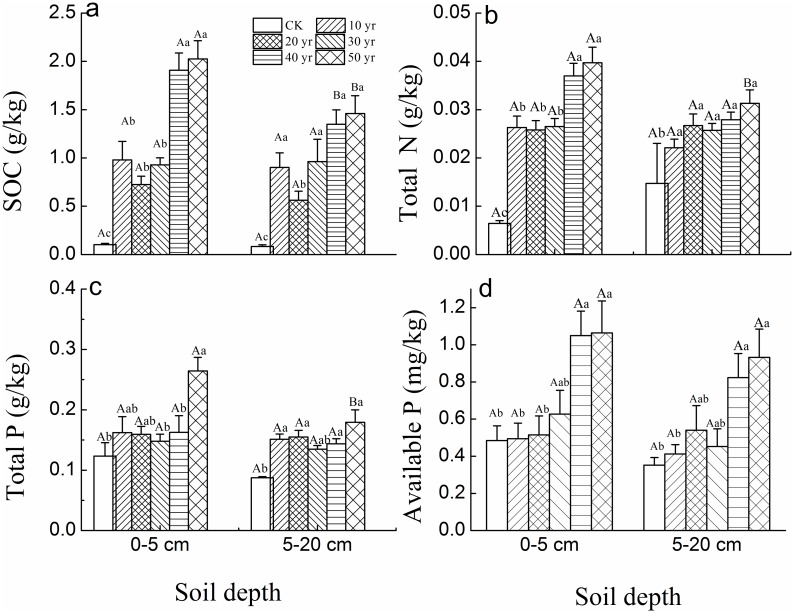
Mean concentration of the SOC, total N and total and available P (mean ± SE) for *H*. *ammodendron* plantations planted in different decades across 50 years (CK-mobile sand dune). (Different uppercase letters indicate significant differences in soil properties between two soil depths in the same plantation age at *p*<0.05. Different lowercase letters indicate significant differences in soil properties among different plantation ages with the same soil depth at *p*<0.05).

SOC in 40 and 50-year-old sites in both topsoil and sub-topsoil layers of *H*. *ammodendron* plantations was much higher (respectively 2 and 1.5 g/kg) than corresponding soil layers in the mobile sand dune (~0.1 g/kg) (Fig 4a and 4b). Additionally, in 40 and 50-year-old plantation topsoil, SOC, total N and available P were significantly larger than younger plantations by around 100, 50 and 100% respectively (Fig 4a, 4b and 4d), in contrast, the lowest values were recorded in mobile sand dune. However, the only significant differences between the two soil layers occurred for SOC in 40 and 50-year-old plantations and for N, total P in 50-year-old plantation only.

**Fig 5:**
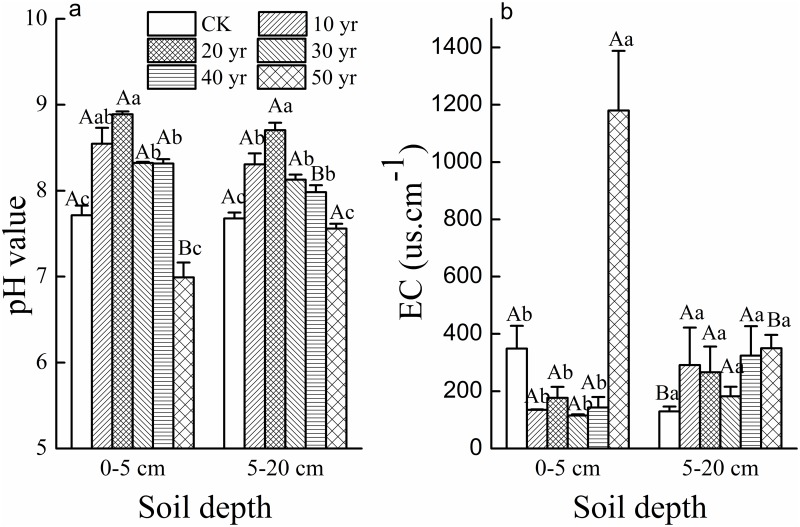
Soil pH value (a) and electrical conductivity (EC us.cm^-1^) (b) (mean±SE) in the 0-5cm and 5-20cm soil layers of *H*. *ammodendron* plantations planted in different decades across 50 years (CK-mobile sand dune). (Different uppercase letters indicate significant differences in soil properties between two soil depths in the same plantation age at *p*<0.05. Different lowercase letters indicate significant differences in soil properties among different plantation ages with the same soil depth at *p*<0.05).

Topsoil pH was significantly more alkaline in plantations in 10-20-year old plantations (pH 8.5–8.8) than in CK (pH 7.7) and the older plantations (Fig 5a). Although pH declined in the 30 and 40-year-old plantations, it remained moderately alkaline at pH 8.3 whereas it became neutral (pH = 7.0) in 50-year-old plantations. Subsoil pH was similar, however the 40-year-old sites were significantly less alkaline in comparison to the topsoil values, while the 50-year-old sites were significantly more alkaline (Fig 5a). Mobile sand dune pH was similar to the 50-year-old sites and was also significantly more neutral than the younger plantations. Topsoil EC in 50-year-old sites was significantly larger than younger plantations and CK (Fig 5b). Additionally, EC of the oldest site was significantly larger in the topsoil by a factor of four. EC marginally increased with plantation age in the subsoil, however the change was not significant (Fig 5b).

### Changes in soil crusts

The thickness of soil crusts increased continually from 0.25 cm in the 10-year sites to over 3cm thick in the 50-year sites, signifying the gradual stabilization of the mobile sand dunes (Table 1). That is, the ground surface in the mobile sand dune transitioned from non-crust (mobile sand dune) to a physical crust. Physical crusts transitioned to a combination of physical and biological soil crusts (BSCs) in the 30-year sites and to BSCs, which dominated in the 50-year sites. Physical crust coverage peaked after 20 years, then decreased as BSCs cover increased over the following decades, to a peak coverage of 53.9% in the 50-year-old site.

**Table 1:** Changes in soil crust characteristics on mobile sand dunes (CK) and of *H*. *ammodendron* plantations established in each decade over 50 years.

Plantation age (years)	Crust type	Crust cover (%)	Crust & topsoil thickness (cm)
**CK**	None	0	0
**10**	Physical crust	39.11–50.2	0.25–0.75
**20**	Physical crust	49.93–63.1	0.75–1.35
**30**	Physical crust + BSCs	40.34–45.22 + 11.22–29.53	1.35–1.80
**40**	Physical crust + BSCs	8.64–28.54 + 40.23–42.06	1.80–2.30
**50**	BSCs	51.0–67.12	2.30–3.30

### Development of herbaceous vegetation in *H*. *ammodendron* plantations

Though the Shannon-Wiener, Simpson indices and number of plant species among plantation ages were similar (Table 2), the perennial plant *Limonium aureum* emerged following 30-year-old plantations. The total herbaceous coverage significantly increased over time after establishment of *H*. *ammodendron*. Coverage peaked at 57% in 50-year-old sites, which was significantly different from that of the 10, 20 and 30-year-old plantations, while not from 40-year-old plantations (Table 2).

**Table 2:** Herbaceous species diversity indices, coverage and species number (mean±SE) of life-form in *H*. *ammodendron* plantations established each decade over 50 years.

Plantation age (years)	Shannon-Wiener index	Simpson index	Herbaceous coverage	Species number of plant life-form	
				Annual	Perennial
**10**	0.96±0.19 a	0.51±0.11 a	0.09±0.06 c	4	0
**20**	0.62±0.14 a	0.49±0.08 a	0.11±0.02 c	4	0
**30**	1.27±0.19 a	0.67±0.07 a	0.16±0.07 b	3	1
**40**	1.36±0.19 a	0.70±0.05 a	0.45±0.18 ab	5	1
**50**	1.47±0.20 a	0.69±0.02 a	0.57±0.14 a	4	1

### Canonical correspondence analysis between vegetation composition and soil properties

**Fig 6:**
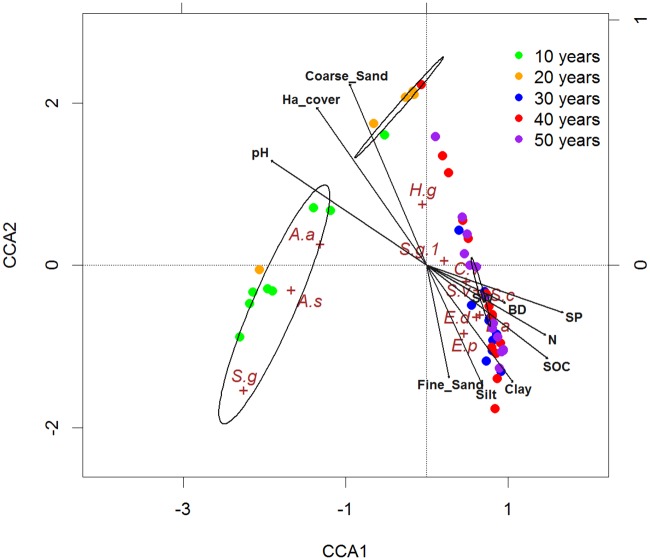
CCA ordination of plantation plots and native herbaceous distribution in relation to soil properties and coverage of *H*. *ammodendron* across 5 decades. (Circles show the plots and plus signs show the species’ relative abundance; *A*.*s*, *Agriophyllum squarrosum; A*.*a*, *Artemisia arenaria; C*.*v*, *Chloris virgate; S*.*v*, *Setaria viridis; S*.*c*, *Salsola collina; E*.*d*, *Echinopilon divaricatum; E*.*p*, *Eragrostis pilosa*; *H*.*g*, *Halogeton glomeratus*; *S*.*g*, *Stipa glareosa*; *L*. *a*, *Limonium aureum*).

CCA results showed the first axis (CCA1) alone explained 44.4% of total variance. Taken together, (CCA2) data set explained 64.3% of total inertia, indicating a relative high vegetation composition-soil environment correlation. The relative contribution of each environmental variable to CCA1 and CCA2 is illustrated in the CCA bi-plot (Fig 6). On one hand, the clay (r = 0.76, p<0.001), silt (r = 0.62, p<0.01), SOC (r = 0.89, p<0.001), Total N (r = 0.93, p<0.001) and Available P (r = 0.97, p<0.001) all showed strong positive correlation with CCA1 axis, whereas coarse sand (r = -0.59, p<0.001) and pH (r = -0.91, p<0.001) showed strong negative correlation along CCA1 (Fig 6). The CCA ordination procedures indicated that clay and silt content had a significant positive correlation with SOC, total N, total P and Available P, and exhibited a negative correlation with pH and coarse sand (Fig 6). Increasing clay and silt content also had a slightly positive correlation with soil water content and bulk density, although correlations were not significant. On the other hand, the CCA indicated that herbaceous species distribution was dependant on soil property gradients and increasing plantation age. Therefore, the plants clustered in three habitats, which generally coincided with decadal plantations. In the youngest plantations (10-year-old), the herbaceous vegetation (green circles) was clustered to the lower left of the CCA plot, which typifies an ongoing arid environment. This herbaceous cluster was clearly separated from the 20-year-old plantations (orange circles) where vegetation was clustered in the upper left centre of the CCA bi-plot. Although these habitats were both correlated with coarse sand, the strong influence of *H*. *ammodendron* growth impacted on the incidence of the relevant species in each plantation. Herbaceous plants in the older plantations (30-, 40- and 50-year-old plantations) were largely clustered in the right centre portion of the CCA plot (Fig 6) where the most influential environmental gradients were increasing clay content and SOC.

There were 11 individual herbaceous species across the five decadal plantations, where each decadal plantation had a unique representation of species, which the CCA bi-plot clearly defined (Fig 6). However, *Halogeton glomeratus* was an exception with a relative abundance of 0.46 indicating it was represented in each decadal plantation. The 10-year-old plantation was dominated by pioneer sand species of *Agriophyllum squarrosum* and *Artemisia arenaria* at 2.1 and 0.4 relative abundance respectively. The 20-year-old plantation was dominated by *H*. *glomeratus at* 8% relative abundance, with *A*. *squarrosum* and *A*. *arenaria* reduced to 1.3% and 0.4% respectively. The older plantations’ vegetation cluster comprised of; *Chloris virgata*, *Setaria viridis*, *Salsola collina*, *Echinopilon divaricatum*, and *Eragrostis pilosa*. These herbaceous species were restricted to environments that were influenced by increasing SOC, clay, silt and improved soil nutrition. *E*. *divaricatum* and *E*. *pilosa* were the lead species on the oldest of the plantations (40- and 50-year-old), with a relative abundance value of 26.7% and 115.2% for *S*. *collina*, and 28.5% and 27.2% for *S*. *viridis* respectively (Fig 6).

## Discussion

### Development of *H*. *ammodendron*

According to Gregory *et al*. [36], Zhao *et al*. [10] and Li *et al*. [18, 19] plantation shrubs as those used in desert reclamation, could reduce wind erosion on dune surfaces and enhance dust fall and deposition. This in turn facilitating the colonization and development of BSCs, as well as providing protection to soil crust and herbaceous species. Thus healthy and sustained long term growth of plantation shrubs are important factors in determine whether mobile sand dunes are stabilized and ecological restoration is achieved. This current study demonstrated that *H*. *ammodendron* could form a sparse, but reasonably stable plantation community with sustained growth in a very arid environment over 50 years. Conversely, Li *et al*. [19] indicated that other shrub species began to degrade after 20 years. These initial woody species were gradually replaced by shallow-rooted native herbaceous plants due to drought. One of the key factors in responding to this drought is increased BSCs coverage [19, 20], as many studies demonstrated BSCs could reduce rain permeability in soil in arid and semiarid zones [37, 38]. This could explain why soil water content at depth were much lower in older than in younger vegetation areas. In contrast, soil water content at 0-20cm soil depth was not reduced in 50-year-old plantations probably because BSCs could retard evaporation and conserve soil moisture for longer under low rainfall due to its fine texture [39]. Indeed, Brotherson and Rushforth [40], found that the formation of biological soil crust provides a compact protecting coat on the soil surface, which lowers the soil surface evaporation. Ground water is generally unavailable as it is too deep for these shrubs, so precipitation is the only source of water for *H*. *ammodendron* growth in these plantation sites. Accompanied with the development of *H*. *ammodendron* during the process of sand dune stabilization, deep soil water content tends to be lower in older vegetation areas than in younger vegetation areas due to high evapotranspiration and low recharge. Additionally, we can consider similar climatic conditions for the different plantation age groups after *H*. *ammodendron* planting. Because analysis of the metrological data by Zhu et al. [41] from 1961 to 2009 in Minqin desert area showed no significant increasing trend for mean annual precipitation during the past 50 years, thus precipitation did not contribute to higher leaf development and coverage of *H*.*ammodendron* in the initial 20 years plantations in comparison with the older sites. In this sense, the variation of *H*. *ammodendron* cover which peaked in 20- year-old plantations and then decreased with plantation age, could be explained by decreasing available soil water at depth due to lower soil permeability to rain. However, the cover reduction in our study was much less than other shrubs planted in the Shapotou region for example, where after 50 years cover was reduced from 33% to 9% and which was associated with a development of micro-biotic crusts (BSCs) and reduced soil water contents at depths [18]. *H*. *ammodendron* is a significantly longer-lived shrub species and is well suited to the arid desert regions of western China, even though in our study it lost 30% coverage over 5 decades.

### The development of soil properties of the *H*. *ammodendron* shrublands with plantation age

Many studies from semiarid and arid areas worldwide have reported the recovery of topsoil characteristics through establishing sand-binding vegetation in a sand-burial environment [2, 3, 11, 18, 20, 34, 42]. Our results indicate that in the surface soil (0-5cm) or sub-surface soil (5-20cm), the soil conditions improved following *H*. *ammodendron* shrub establishment. These changes included an increase in clay and silt content, as well as soil organic C, total N and available P with increasing *H*. *ammodendron* plantation age. Similar changes were observed in other restoration studies in Northern China [11, 31] which were attributed to the capture of fine aeolian particles and their deposition amongst the shrubs. Increases in C and N concentrations in the soil depend less on soil parent material [43] but are reliant on deposition and subsequent vegetation activity. In the older plantations (40- and 50-year-old), BSCs and herbaceous developed abundantly, while the capture of some plant litter (not measured) may have contributed to improve the soil nutrition. Meanwhile, the abundant BSCs and herbaceous plants assisted in the further capture of fine soil particles thus contributing to improve soil physical conditions. Whereas total P concentration is reliant on weathering processes and nutrient cycling of P [44] hence increases of P in 10-50-year *H*. *ammodendron* plantations was not apparent. Meanwhile, EC was generally stable and low, with the exception of 50-year-old plantation where the continual extraction of water enriched soil salt in this low rainfall environment. The *H*. *ammodendron* is a succulent halophyte which can uptake amounts of salt ion from the soil in their growth [45], which did not present issue for shrub longevity. The changes in pH in our study were parabolic in shape, whereas other studies indicated a linear increase in pH for the same period. In the initial 20 years, our revegetation methods resulted in an increase in pH, which corresponded to, but was more pronounced than in other studies of Wang *et al*. [20] and Li *et al*. [18] for the same period. We attributed this to the initial extensive soil disturbance during plantation development and corresponding loss of organic material and H^+^ ions through oxidizing processes. In these medium to highly alkaline conditions, availability of macronutrients such as N and P was low and limited plant establishment and restoration sustainability. Following 40 plus years of deposition, vegetative activity and the development of BSCs, pH eventually moved into a neutral range (pH 7) where macro and micronutrients were more readily available. Consequently, in these latter plantations, there was acceleration in herbaceous coverage and the further development of BSCs.

According to results from Li *et al*. [3, 18] and Wang *et al*., [20] over a 50-year period in Shapotou desert, changes in soil properties and vegetative features were more rapid in the early stages than in the latter stages of dune stabilization. Su and Zhao [14] in Horqin region also noted a faster restoration rate during the early establishment stage (0–13 years) and a slower rate during the latter successional stage (13–28 years). The recovery rate of soil properties following establishment of *H*. *ammodendron* shrub in Minqin desert mobile dunes in our study, was considerably slower than these previous studies conducted in other desert regions of northwestern China. Here in Minqin, three distinct phases occurred in the chrono-sequence of PSD changes (clay, silt and course sand) and soil nutrition (SOC, and total N) improvement: i) an initial fast development from 0 to 10 years, ii) a stabilizing phase from 10 to 30 years followed by iii) a relatively marked restoration development in 40 and 50-year-old plantations. The rapid increase in clay, silt, SOC and total N between 0 and 10 years whereas there is no change between 10 and 20 year plantations, was mainly due to the planting operation as it played an important role in the initial restoration stages. Most of straw checkerboard used to stabilize the dunes during the shrub planting were blow away due to heavy wind erosion on windward slope. However, a little straw held by soil when firstly planting remained to decompose into the surrounding soil and thus improving soil conditions and supporting growth in early plantations. In later plantations, the influence of straw largely diminished and can be neglected.

Although plant litter, plant root activity and soil crust development can improve topsoil properties, the effects of these on soil physical and chemical properties to depth are limited [10, 11]. Our results correspond with these previous studies in Inner Mongolia, however we found significant responses in PSD and soil nutrients with increasing soil depth during this restoration process in 40 and 50-year-old plantations, as shown in the canonical coordinate analysis. In arid and semiarid rangelands in northern China, such as the Shapotou area [3, 21] and the Horqin sand lands [14], where these studies found that most of the changes in soil physio-chemical properties mainly occurred in the topsoil but were less marked with increasing soil depth. These differences in restoration time we attributed to the differences in aridity, where Horqin is semiarid and Shapotou is in a transitional semiarid area, in which they both receive, on average 2–3 times more rainfall than the Minqin region.

### Vegetation characteristics and soil crust development

Previous studies have demonstrated that during mobile sand dune stabilisation the previous dominant native plant communities were relatively quickly replaced by shallow-rooted herbaceous species due to changes in the soil moisture and development of BSCs [19, 20]. As a result, Wang *et al*. [20] reported that in five years the annual species that formerly grew sporadically in the migrating desert dunes had disappeared. The pattern of change corresponded to our study, but at a much slower rate. This was due to the gradual initial development of physical soil crusts through 2 decades and the eventual development of 3.3 cm thick biological soil crusts with 57% ground cover after 50 years. The formation and development rate of BSCs in our study indicated that in 10 and 20-year-old plantations physical crusts had formed, however BSCs only sparsely appeared in 30-year-old plantations. In contrast, BSCs (consisting of cyanobacteria and algae crust) dominated the landscape after 27 years of revegetation in the Shapotou area [19]. The ground surface was covered by 70% moss crust in 15-year-old plantations located in the Horqin Sand Land [12], which was also the case in the Tengger Desert [19]. Consequently, Li *et al*. [18] recorded 15 herbaceous species after 50 years of revegetation in the Shapotou region. Also in Horqin Sand Land stabilized dunes the dominant herbaceous plants; *Corispermum elongatum*, *Melissitus ruthenicus* and *Cleistogenes squarrosa* emerged within 20-years [2]. However, in our study, the herbaceous species diversity, the number of herbaceous species and the succession rate of vegetation in Minqin was limited, we recorded only five herbaceous species in the 50-year-old plantations, and in 30-year-old plantations the emergence of BSCs had only just begun. In the 40-year-old plantations BSC coverage had increased to ~44% facilitating the distribution and dominance of *E*. *divaricatum* and *E*. *pilosa* consequently contributed to the development of future native herbaceous vegetation.

Soil characteristics are key drivers in plant community succession [46, 47], meanwhile plant community succession processes reflect the process of the individual plant adapting to the soil conditions [48]. Numerous studies have demonstrated that soil texture and nutrition in arid ecosystem topsoils are beneficial to herbaceous plants [3, 49–51]. Our CCA results demonstrated that herbaceous development was closely associated with the changed soil texture (increased clay and silt percentage) and availability of soil nutrients. The 10- and 20-year-old plantations and their respective vegetation; *A*. *squarrosum*, *A*. *artemisia* and *H*. *glomeratus*, were closely related with high coarse sand content and low nutritional requirements. The changes in topsoil properties attributed to the interaction between plants and the soil crust by Zhao *et al*. [11] were apparent in the 40-year-old plantation, which gave rise to increased soil nutrition and finer soil texture in a habitat consisting of higher relative abundance values of *E*. *divaricatum* and *E*. *pilosa*. The change in habitat driven by the significant increases in herbaceous coverage after 30 years of stabilization and the rise of BSCs may contributed to decreasing pH, which corresponds to studies by Su and Zhao [14] in northeast China, Horqin Sandy Land. Tornquist *et al*. [52] also found decreasing trends in pH due to the secretion of organic acids and the release of CO_2_ from roots and/or micro-organisms, with increases in herbaceous cover.

Consequently, after 5 decades since the shrub *H*. *ammodendron* was established in the Minqin desert region, the eco-environment had improved. However, the restoration rate was much slower than reported elsewhere in northern China sand dune ecosystems. Prominent factors that influence the rate of ecosystems recovery in most arid areas the amount of rain and number of rain events [53]. Therefore, we attributed the slower desert restoration and sand dune stabilisation in the Minqin region to infrequent and low precipitation (110 mm). In comparison, the semiarid Horqin Sand Lands of northeast China which receives 365 mm annually [11], while the Shapotou region located in a transitional zone between arid and semiarid climate in northwest China, receives 220 mm annually.

### Suggestions for plantation management and ecological restoration

Our results indicated that *H*. *ammodendron* is a suitable species for successful ecological restoration in Minqin desert zone and it highlighted that in arid desert ecosystems plantation longevity is essential for the deposition and accumulation of aeolian soil components and the development of BSCs. The formation period of biological soil crusts requires increasingly more time with the level of aridity, therefore the shrub species selected for afforestation are likely to require extended maintenance to sustain the formation of large quantity of BSCs and herbaceous plants over an extended period. Thus, the key species selection criteria are longevity and aridity tolerance. At small spatial scales the change in soil resources and conditions are important determinates of vegetation features [54, 55]. Therefore, in order to stabilize the mobile sand dune through the development of natural herbaceous cover under the protection of a plantation, it is vital to increase maintenance and protection of existing reserves. Avoidance of topsoil damage and environmental protection should include the exclusion of grazing animals and other destructive activities, which is likely to be more important than additional restoration work.

## Conclusion

Establishment of *H*. *ammodendron* promoted the formation and development of topsoil, soil crusts and herbaceous community succession; all be it over an extended period of 5 decades. The development of herbaceous species ground cover, biological soil crusts and improved physical and chemical soil properties in our study area were very slow, compared to other northern China afforestation sites: the most likely plantation age for marked ecological restoration improvement of soil crust, soil nutrients, and herbaceous occurred at the 50 years, while the development and difference among initial 40 years of plantation were usually non-significant. Herbaceous development was closely associated with the increasing restoration age, finer soil texture and availability of soil nutrients. Underpinning the relatively slower afforestation of the Minqin desert was very low precipitation. Overall, our results highlighted that in order to successfully revegetate a desert area, long-lived halophyte shrub species are key to soil development, understory re-vegetation and suppressing renewed desertification.
